# Mutation Types of CYP71P1 Cause Different Phenotypes of Mosaic Spot Lesion and Premature Leaf Senescence in Rice

**DOI:** 10.3389/fpls.2021.641300

**Published:** 2021-03-23

**Authors:** Yuhan Zheng, Jiangmin Xu, Fujun Wang, Yongchao Tang, Zheng Wei, Zhiyuan Ji, Chunlian Wang, Kaijun Zhao

**Affiliations:** ^1^National Key Facility for Crop Gene Resources and Genetic Improvement, Institute of Crop Sciences, Chinese Academy of Agricultural Sciences, Beijing, China; ^2^Institute of Rice Research, Guangdong Academy of Agricultural Sciences, Guangzhou, China

**Keywords:** lesion mimic mutants, leaf senescence, reactive oxygen species, cell death, mutation types, rice

## Abstract

Lesion mimic mutants (LMMs) are ideal materials for studying programmed cell death and defense response in plants. Here we report investigations on two LMMs (*msl-1* and *msl-2*) from the indica rice cultivar JG30 treated by ethyl methyl sulfone. Both of the mutants showed similar mosaic spot lesions at seedling stage, but they displayed different phenotypes along with development of the plants. At tillering stage, larger orange spots appeared on leaves of *msl-2*, while only small reddish-brown spots exhibit on leaves of *msl-1*. At heading stage, the *msl-2* plants were completely dead, while the *msl-1* plants were still alive even if showed apparent premature senility. For both the mutants, the mosaic spot lesion formation was induced by light; DAB and trypan blue staining showed a large amount of hydrogen peroxide accumulated at the lesion sites, accompanied by a large number of cell death. Consequently, reactive oxygen species were enriched in leaves of the mutants; SOD and CAT activities in the scavenging enzyme system were decreased compared with the wild type. In addition, degraded chloroplasts, decreased photosynthetic pigment content, down-regulated expression of genes associated with chloroplast synthesis/photosynthesis and up-regulated expression of genes related to senescence were detected in the mutants, but the abnormality of *msl-2* was more serious than that of *msl-1* in general. Genetic analysis and map-based cloning revealed that the lesion mimic and premature senescence traits of both the mutants were controlled by recessive mutated alleles of the *SL* (Sekiguchi lesion) gene, which encodes the CYP71P1 protein belonging to cytochrome P450 monooxygenase family. The difference of mutation sites and mutation types (SNP-caused single amino acid change and SNP-caused early termination of translation) led to the different phenotypes in severity between *msl-1* and *msl-2*. Taken together, this work revealed that the CYP71P1 is involved in regulation of both premature senescence and cell death in rice, and its different mutation sites and mutation types could cause different phenotypes in terms of severity.

## Introduction

Plant lesion mimic mutants (LMMs) spontaneously forms necrotic spots on leaves, leaf sheaths and stems under the external conditions without damage or pathogen infection (Hu et al., 1996). LMMs have been identified in a wide range of plants, including maize (Walbot, 1991), *Arabidopsis thaliana* (Dietrich et al., 1994), wheat (Yao et al., 2009), barley (Wolter et al., 1993), rice (Takahashi et al., 1999), and peanut (Badigannavar et al., 2002).

The typic phenotype of a LMM is similar to the hypersensitive response of plant, which is a rapid response to invasion of pathogens, characterized by the rapid death of local cells at the invasion sites. The hypersensitive response can limit growth of microorganisms (Lorrain et al., 2004), usually accompanied by the characteristic of broad-spectrum disease resistance. Therefore, LMMs are ideal materials for studying programmed cell death and defense response mechanisms of plants (Gao et al., 2019).

There are various genetic pathways underlaying the lesion mimic phenotypes of rice. So far, more than 20 genes associated with rice lesion mimic have been cloned, including *spl7* (Yamanouchi et al., 2002), *spl11* (Yin et al., 2000; Zeng et al., 2004), *spl33* (Wang et al., 2017), *nls1* (Tang et al., 2011), and *sl* (Fujiwara et al., 2010). Most of the lesion mimic traits are controlled by a single recessive gene derived from mutation of cell-death negative regulators (Huang et al., 2010). For example, *SPL7* encodes a heat-stress transcription factor. The mutant allele *spl7* with a single base substitution in the coding region of a DNA binding domain, resulting in the conversion of highly conserved amino acid from tryptophan to cysteine, leads to change of the coded protein function (Yamanouchi et al., 2002). *SPL11* encodes the U-box/arm protein SPL11 with U-box domain-dependent E3 ubiquitin ligase activity. A single base substitution in the first exon of the mutant allele *spl11* results in the early termination of translation, which confers broad-spectrum resistance to rice blast and bacterial blight (Yin et al., 2000). Likewise, *SPL33* encodes a eukaryotic translation extension factor eEF1A. A single base mutation in the mutant *spl33* results in the early termination of translation, resulting in loss of protein function and activation of defense response to rice blast and bacterial blight (Wang et al., 2017). *NLS1* encodes a typical CC-NB-LRR type protein, its mutant *nls1* exhibits constitutive defense responses, including cell death, excessive accumulation of hydrogen peroxide and salicylic acid (SA), and enhanced resistance to bacterial pathogens *Xanthomonas oryzae* pv. *oryzae* (*Xoo*) (Tang et al., 2011). Furthermore, *SL* encodes the CYP71P1 protein of cytochrome P450 monooxygenase family. Mutation of *SL* causes the so called Sekiguchi lesion (Fujiwara et al., 2010). In addition to the aforementioned mutants, the *spl30* mutant also increased the resistance to rice blast (Ruan et al., 2019), the *cdr1* significantly increased rice blast resistance (Takahashi et al., 2003), and the *spl28* plants showed enhanced resistance to both blast and bacterial blight (Qiao et al., 2010). Collectively, most of the rice LMMs show improved resistance to pathogens (Xu et al., 2018; Tian et al., 2020).

Leaves are the typical photosynthetic organs of plants (Lohman et al., 1994). Normal leaf senescence is a spontaneous physiological process of plant development to a certain stage, usually accompanied by the redistribution of photosynthetic products (Buchanan-Wollaston, 1997; Lim et al., 2007). The premature senescence of plant leaves is related to changes of cell physiological and biochemical features (Breeze et al., 2011), such as degradation of macromolecular substances (proteins and nucleic acids) (Thompson et al., 1998), severe degradation of chloroplasts and decrease of chlorophyll content (Lee et al., 2009), and membrane lipid peroxidation due to the explosion of reactive oxygen species (Thompson and Lake, 1987). Premature senescence of rice shortens the functional period of leaves and seriously affects grain development during and after grouting, resulting in significant decrease of yield and quality (Gregersen et al., 2013). It has been reported that 1-day delay in leaf senescence could increase rice yield by about 1% (Liu, 1983). Therefore, analysis of the molecular regulation mechanism underlying rice leaf senescence is of great significance for development of elite rice germplasms and breeding super high yield rice varieties.

The process of premature or early senescence can be divided into three stages: the initial stage, the decline stage and the end stage (Noodén et al., 2010). Generally, the senescence-associated genes (SAGs) were classified into three types. The first type is down-regulated genes, which are characterized by significantly reduced mRNA level in senescent leaves, or their expression was inhibited during leaf senescence. The second type refers to those genes with strong senescence specificity, which are activated during senescence, but not expressed at other times. The third type SAGs are similar to the second type genes, but they have a low transcription level in early leaf development, and the transcription level elevates by leaps and bounds during senescence (Gan and Amasino, 1997). Moreover, there are some other SAGs that are specifically expressed during senescence, whose mRNAs can be detected only when the leaf is senescent. For example, three senescence-specific expression genes (*Osl20*, *Osl85*, and *Osl295*) have been cloned (Lee et al., 2001). These genes are involved in amino acid metabolism, fatty acid metabolism and protein degradation, thus affecting the senescence of rice leaves.

It has been reported that LMMs are associated with premature senescence (Qiao et al., 2010; Lee et al., 2018). For example, the growth vigor of mutant *lmm24* is obviously weaker than that of the wild type (Zhang et al., 2019). In present study, we identified two LMMs, designated as mosaic spot lesion mutants *msl-1* and *msl-2*, from indica rice cultivar JG30 treated by ethyl methyl sulfone (EMS). We also observed that *msl-1* and *msl-2* showed a characteristic pattern of premature senescence in addition to the lesion mimic phenotype, but the molecular mechanism underlaying these mutant phenotypes remains unknown. After systematic identification of the phenotypes and physiological characteristics of the two mutants, genetic analysis and map-based cloning of the genes underlying the mutant phenotypes were carried out. We found that the phenotypes of *msl-1* and *msl-2* were controlled by mutated alleles of the so-called *SL* (Sekiguchi lesion) gene (Fujiwara et al., 2010). The phenotype difference between *msl-1* and *msl-2* is caused by the different mutation sites and types in the *SL* gene. This study demonstrated that mutation of *SL* not only mediated programmed cell death of rice, but also led to premature senescence, and mutation sites and types could cause different phenotypes in terms of severity. Based on previous publications and our present findings, a working model for *SL*-involved rice leaf senescence and cell death was proposed.

## Materials and Methods

### Plant Materials

The LMMs *msl-1* and *msl-2* were isolated through EMS treatment of the indica rice cultivar JG30. After multiple generations of continuous selfing, mutant lines with stable phenotypes were selected from their progenies. The *msl-1* and *msl-2* plants were crossed with japonica rice cultivar 02428, respectively, the phenotypes of F_1_ and segregation ratio of F_2_ plants were surveyed for genetic analysis and gene mapping. All the rice materials were grown by conventional culture in the net room of Institute of Crop Science, Chinese Academy of Agricultural Sciences (Beijing). From seedling stage to mature stage, the phenotypic status of the mosaic spot lesions have been observed and recorded. At mature stage, 10 individual plants were randomly selected from each mutant line to investigate their traits of plant height, number of tillers, panicle length, effective panicles, seed setting rate, 1,000-grain weight, grain number per panicle, filled grain number per panicle, heading time, primary branch and secondary branch numbers per panicle. The two-tailed Student t-test was used to compare the agronomic traits of the mutants and wild-type plants.

### Shading Experiment

Due to the uncertainty of the location of the lesion mimic spots, the top and fully expanded leaves of the mutant plants with non-spotted phenotype were wrapped with tinfoil at tillering stage, which were continuously shaded for 1 week, and occurrence of the mosaic spots was recorded by taking photos. After that, the tinfoil was removed and light was restored. A week later, occurrence of leaf lesions was observed and photographed.

### Histochemical Analysis

#### Trypan Blue Staining

According to the method of Yin et al. (2000), plants of the wild-type JG30 and mutants *msl-1* and *msl-2* with the same growth vigor were selected at tillering stage. In brief, leaves at the same part of selected plants were cut off and placed in a 15cm long glass tube, then trypan blue staining solution was added and boiled for 10 min. After kept in dark for more than 12 h, the leaves were transferred to 25 mg/ml chloral hydrate to decolorize for 3 days. Blue spots on the leaves were recorded and photographed.

#### DAB (3,3′-Diaminobenzidine) Staining

According to Thordal Christense’s method (Thordal-Christensen et al., 1997). Briefly, leaves of the mutants and WT at the same time were soaked in 1 mg/ml DAB (pH = 5.8) solution and dyed for more than 8 h under dark conditions. The leaves were taken out and decolorized in boiling water bath with 95% alcohol for 10 min, fresh anhydrous alcohol was replaced to decolorize until the leaves were transparent. Then observed whether there are reddish brown spots on the leaves of the mutants, and photographed.

### H_2_O_2_, MDA Content and ROS-Scavenging Enzyme Assays

At peak tillering stage, leaves of JG30 and the mutants were taken to prepare tissue homogenate for determination of hydrogen peroxide (H_2_O_2_) and catalase (CAT), total superoxide dismutase (T-SOD), and malondialdehyde (MDA). Three biological replicates were measured for each sample. All procedures were according the manuals of reagents purchased from Nanjing Jiancheng Bioengineering Institute.

### Determination of Photosynthetic Pigment Content

Leaves of wild-type JG30 and the mutant *msl-1* and *msl-2* at tillering stage were collected to measure photosynthetic pigment. The specific operation steps are as follows: weighed about 0.01 g leaves without midrib, cut leaves into pieces, then added 5 mL of 95% ethanol, placed in a 4°C refrigerator to keep out of light, soaked for 24 h and shaken every 8 h until the leaves are completely discolored. Three biological replicates were measured for each sample. The absorbance values at 470, 649, and 665 nm were measured by spectrophotometer with 95% ethanol as blank control. The photosynthetic pigment content (mg/g) were calculated according to the methods of Lichtenthaler (1987). The calculation formula of photosynthetic pigment content (mg/g) is as follows: Chla = (13.95⋅Abs665–6.88⋅Abs649) V/(1,000⋅m); Chlb = (24.96⋅Abs649–7.32⋅Abs665) V/(1,000⋅m); Car = (1,000⋅Abs470–2.05⋅Chla–114⋅Chlb)/245 × V/(1,000⋅m); Total Chl = Chla + Chlb. In the above formula: V: total volume of chlorophyll extract (ml); m: fresh weight of material (g).

### Transmission Electron Microscopy (TEM) Assay

At tillering stage, leaves of the wild type and mutants were fixed in 2.5% pre-cooled glutaraldehyde for 24 h, then the samples were rinsed with 0.1 mmol/L phosphoric acid buffer (pH = 7.2) for three times, each time for 10 min. After washing, the samples were fixed with 1% osmic acid stationary solution (pH = 7.3) for 4 h; after preliminary fixation, the samples were dehydrated by gradient ethanol, and then embedded in epoxy resin after dehydration; After that, the slices were stained with uranyl acetate and lead citrate, observed and photographed under the transmission electron microscope (Hitachi, Japan).

### RNA Extraction and Quantitative Real-Time PCR (qRT-PCR) Analysis

Total RNAs were extracted by Trizol method (Invitrogen, United States). The treated RNA samples were reversely transcribed into cDNA by reverse transcription Kit (TIANGEN, Beijing, China). The SYBR^®^ Premix ExTaqTM II kit was used to configure the reaction system, and ABI 7500 Real-Time PCR system was used as the PCR quantizer. Using rice *OsActin* as internal reference gene, the reaction volume was 20 μL, containing 10 μL 2 × SYBR Green Master Mix, 2 μL cDNA, 0.4 μL 50 × ROX Reference Dye II, 0.8 μL forward and reverse primers (10 μmol/L) and 6 μL RNase-free H_2_O. The PCR procedure was pre denaturation at 95°C for 3 min, 95°C for 5 s, 60°C for 34 s, and 40 cycles. The relative expression was analyzed by 2^–ΔΔCt^ method (Livak and Schmittgen, 2001), and three biological replicates were measured for each sample. The primers for qRT-PCR analysis are listed in Supplementary Table 1. All primers were synthesized by Sangon Biotechnology Co., Shanghai, China.

### Fine Mapping of *msl-1* and *msl-2*

The F_1_ hybrids were obtained by crossing japonica rice cultivar 02428 as female parent and mutants *msl-1* and *msl-2* as male parents, respectively. The F_2_ segregation populations were obtained by selfing of F_1_ plants. Insertion/deletion (InDel) molecular markers (totally, 256) distributed evenly on 12 chromosomes of rice were used to screen the polymorphism of the parents, and then individual F_2_ plants with extreme lesion mimic phenotype were selected for genetic linkage analysis. According to the results of preliminary mapping, new molecular markers were further developed, the mapping populations were expanded and 10 pairs of primers (Supplementary Table 2) were used for fine mapping.

### Analysis of Candidate Genes, Amino Acid Sequences, and Evolutionary Relationship

Based on the RGAP^1^ genome information, we downloaded the sequences of all candidate genes within the mapped location intervals, then used Primer3.0^2^ to design primers to amplify the candidate genes of the wild-type JG30 and mutants. The PCR-amplified products were recovered and sent for sequencing by Sangon Biotechnology Co., Shanghai, China. Sequencing results were analyzed with the DNAMAN software.

The deduced homologous proteins of *msl-1* and *msl-2* in 17 plant species were searched and downloaded from NCBI website through blastx and saved in FASTA format. The multi-sequence alignment software ClustalX was used for the alignment analysis of amino acid sequences. Then MEGA7.0 was used to construct the phylogenetic tree, where we selected neighbor-joining method to construct the tree, and the check parameter Bootstrp was set to 1,000.

### Three-Dimensional Structure Analysis of Proteins

The online protein structure prediction software Swiss-model^3^ was used to conduct homologous modeling for the protein spatial structure of wild-type protein CYP71P1, mutant proteins msl-1 and msl-2, respectively. The protein sequences were saved in PDB format, and the software was used to display, then compared and analyzed the three-dimensional structure of the proteins.

## Results

### Phenotype Identification of *msl-1* and *msl-2*

**FIGURE 1:**
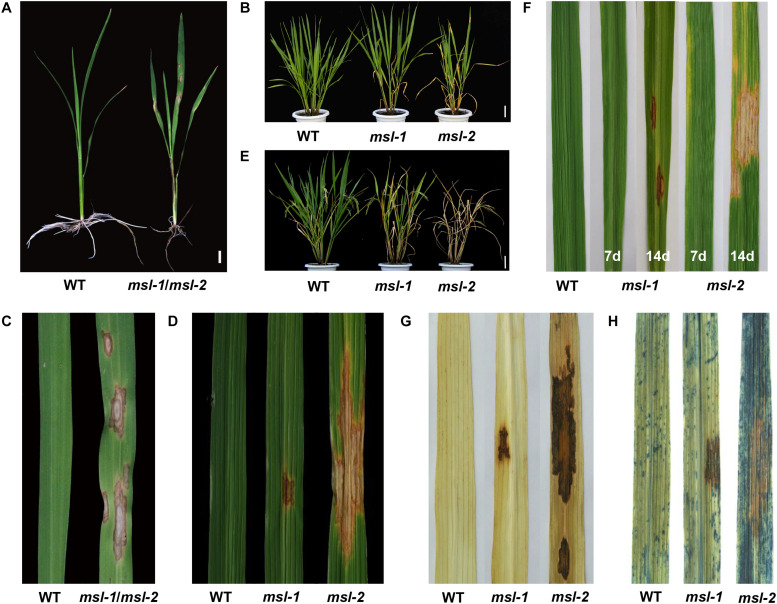
Phenotypic characterization of wild type and mutants. **(A)** Phenotypes at seedling stage (30 days after sowing). **(B)** Phenotypes at the young panicle differentiation stage. **(C)** Leaves at seedling stage. **(D)** Leaves at tillering stage. **(E)** Plant phenotypes at heading stage. **(F)** Formation of mosaic spot lesion is light dependent. From left to right: leaf of wild type, leaf of *msl-1* covered with aluminum foil for 7 days, leaf of *msl-1* recovered to light for 7 days, leaf of *msl-2* covered with aluminum foil for 7 days, leaf of *msl-2* recovered to light for 7 days. **(G)** Diaminobenzidine (DAB) staining showing accumulation of H_2_O_2_ at mosaic lesions. **(H)** Trypan blue staining, showing cell death at the mosaic lesions. Bar = 1 cm **(A)**; Bar = 10 cm **(B,E)**.

Compared with the wild-type JG30, the mutants *msl-1* and *msl-2* showed brown necrotic spots on leaves, leaf sheaths and stems of seedlings at three-leaf stage (25 days post sowing), and the roots displayed dysplasia (Figures 1A,C). At tillering stage, large orange spots appeared on the leaves of *msl-2*, the number of spots increased gradually and finally the spots covered the whole leaf area; while the reddish-brown spots of mutant *msl-1* was small and the number of mosaic spots was relatively less (Figure 1D). At young panicle differentiation stage, the lower leaves of *msl-1* and *msl-2* plants became dying, showing significant premature senescence. In addition, the degree of premature senescence was higher and the number of dead leaves of *msl-2* was more than that of *msl-1* (Figure 1B). At heading stage, the spots on the leaves of *msl-2* appeared in an explosive manner, and the whole plants died, but for *msl-1*, only the lower leaves died and other functional leaves became somewhat yellow compared with the wild-type plants (Figure 1E).

We further investigated agronomic characters of the mutants and WT at maturity stage. Compared with the wild type, plant height, number of tillers, effective panicle number, seed setting rate, 1,000-grain weight, total grains per panicle, filled grains per panicle and secondary branch number of the mutants were all significantly decreased (Table 1). In addition, the wild-type plants started booting about 102 days after sowing, while the *msl-1* plants started booting about 107 days after sowing, and the mutant *msl-2* started booting about 113 days after sowing (Table 1). The development of both *msl-1* and *msl-2* plants were significantly delayed compared with the wild type. In general, the appearance of lesion mimic spots has a more obvious impact on the agronomic traits of mutant *msl-2* (Table 1).

**TABLE 1:** Agronomic traits of the wild type and mutants.

Agronomic traits	Materials		
	WT	*msl-1*	*msl-2*
Plant height (cm)	91.20 ± 6.83	83.29 ± 2.06*	75.67 ± 1.15**
Number of tillers	8.40 ± 1.34	5.29 ± 1.11**	3.33 ± 0.58**
Effective panicle	8.20 ± 1.10	3.57 ± 0.79**	3.00 ± 1.00**
Panicle length (cm)	21.40 ± 1.38	18.98 ± 1.91	19.58 ± 1.29
Grain number per panicle	137.28 ± 15.90	96.56 ± 18.14**	94.72 ± 5.04**
Filled grain number per panicle	116.13 ± 17.38	61.00 ± 11.75**	28.11 ± 2.59**
Primary branch number	11.00 ± 0.76	9.90 ± 0.98	10.28 ± 0.75
Secondary branch number	24.30 ± 5.90	13.16 ± 5.05**	10.83 ± 2.02**
Setting rate (%)	84.35 ± 3.57	63.30 ± 5.22**	29.65 ± 1.64**
1,000-grain weight (g)	26.62 ± 0.17	19.91 ± 0.17**	17.80 ± 0.18**
Heading time (day)	102.60 ± 1.14	107.14 ± 0.90**	113.33 ± 2.08**

The data in table are the average value ± standard deviation of 10 individual plants. * and **, significant differences at P < 0.05 and P < 0.01, respectively (Student’s t-test).

### Light-Induced Response of the Lesion Mimic Mutants

At tillering stage, shading treatment was carried out for the leaves without lesion mimic spots of *msl-1* and *msl-2*. A week later, no spots were found in the area covered by aluminum foil, while mosaic spots appeared in the same age leaves without shading treatment. Interestingly, after the tinfoil removed, and regaining normal light for 7 days, the previously tinfoil-shaded leaves displayed mosaic spots (Figure 1F). These results indicated that formation of the mosaic spots phenotype of *msl-1* and *msl-2* is light dependent.

### Programmed Cell Death and Reactive Oxygen Species Accumulation in the Mutants

Programmed cell death is usually accompanied by accumulation of intracellular reactive oxygen species. In presence of peroxidase, diaminobenzidine (DAB) reacts with H_2_O_2_ to rapidly produce reddish-brown precipitation. According to this, DAB staining was performed for leaves from the mutants and wild-type JG30. Results showed that leaves from both *msl-1* and *msl-2* were stained with reddish-brown polymer deposition, while the WT had no reddish-brown spots (Figure 1G). These observations indicated that growth of the lesion spots was accompanied by the accumulation of a large amount of hydrogen peroxide. Trypan blue is an indicator of cell death. After trypan blue staining, the whole leaf of *msl-2* was stained with dark blue spots, while dark blue spots appeared only on and near to the spots of *msl-1*. Compared with that, the wild-type JG30 did not display obvious dark blue spots (Figure 1H), indicating that programmed cell death had occurred or was occurring in the mutant leaves at tillering stage.

### Premature Senescence of the Mutants

In order to analyze the premature senescence of the mutants in depth, we observed the microstructure of chloroplasts, measured the content of photosynthetic pigment, and determined the expression of genes associated with senescence, chloroplast synthesis and photosynthesis.

**FIGURE 2:**
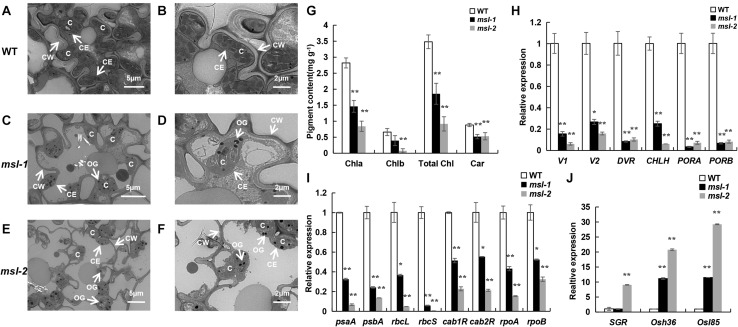
Premature senescence identification of mutant leaves. **(A–F)** Ultrastructural analysis of chloroplasts in wild type and mutants by transmission electron microscopic. C, chloroplast; CE, chloroplast membrane; CW, cell wall; OG, osmiophilic plastoglobuli. **(G)** Determination of photosynthetic pigment content. **(H)** Expression assay of chloroplast synthesis-related genes. **(I)** Expression assay of photosynthetic-related genes. **(J)** Expression assay of senescence-related genes. Error bars represent the standard deviations of three biological replicates. * and **, significant differences at *P* < 0.05 and *P* < 0.01, respectively (Student’s *t*-test).

At maximum tillering stage, leaves from the mutants with lesion mimic spots and the corresponding wild-type leaves were sampled and placed under transmission electron microscopy (TEM) to observe the chloroplast structure. TEM assays showed that the number of chloroplasts in the mesophyll cells of wild type was more, the grana lamella was more abundant; the osmiophilic plastoglobuli was less; and the chloroplast structure was complete (Figures 2A,B). However, in leaves of the mutants, the lamellar structure of thylakoid began to degrade gradually; the vascular structure appeared; the number of osmiophilic plastoglobuli increased significantly; the chloroplast structure was damaged and the chloroplasts began to disintegrate; and the cell wall of mutants were thinner than that of wild type, which may accelerate cell apoptosis and cell structure disintegration in the mosaic spot lesions (Figures 2C–F). Moreover, the damage degree of chloroplasts in *msl-2* was higher than that in *msl-1*, as indicated by more osmiophilic plastoglobuli and more serious chloroplast degradation (Figures 2E,F). The degree of chloroplast degradation was in coincidence with the severity of premature senescence.

The chlorophyll content is an important physiological index to measure the photosynthesis and premature senescence of leaves (Jiao et al., 2012). We found that the contents of chlorophyll a (Chl *a*), chlorophyll b (Chl *b*), total chlorophyll (Total Chl), and carotenoid (Car) in *msl-1* and *msl-2* were significantly decreased compared with those of the wild type at tillering stage (Figure 2G). Notably, the contents of Chl *a*, Chl *b*, and Total Chl in *msl-2* decreased in a larger extent than that of *msl-1* (Figure 2G), suggesting that *msl-2* had a higher degree of premature senescence.

We further examined expression of six chloroplast synthesis-related genes (*V1*, *V2*, *DVR*, *CHLH*, *PORA*, *PORB*), eight photosynthesis-related genes (*psaA*, *psbA*, *rbcL*, *rbcS*, *cab1R*, *cab2R*, *rpoA*, *rpoB*) and three senescence-related genes (*SGR*, *Osh36*, *Osl85*) in the mutants and wild-type by qRT-PCR analysis. Results showed that the expression of six chloroplast synthesis-related genes and all the photosynthesis-related genes in mutant leaves were dramatically down-regulated (Figures 2H,I). In contrast, the expression of senescence-related genes significantly increased in *msl-2* compared with the wild-type. Similarly, the expression of *Osh36* and *Osl85* exhibited elevated expression in the *msl-1* mutant (Figure 2J). Overall, these results suggest that premature leaf senescence occurs in *msl-1* and *msl-2*, and *msl-2* displayed premature senescence in a more serious severity.

### ROS Abnormality in the Mutants

**FIGURE 3:**
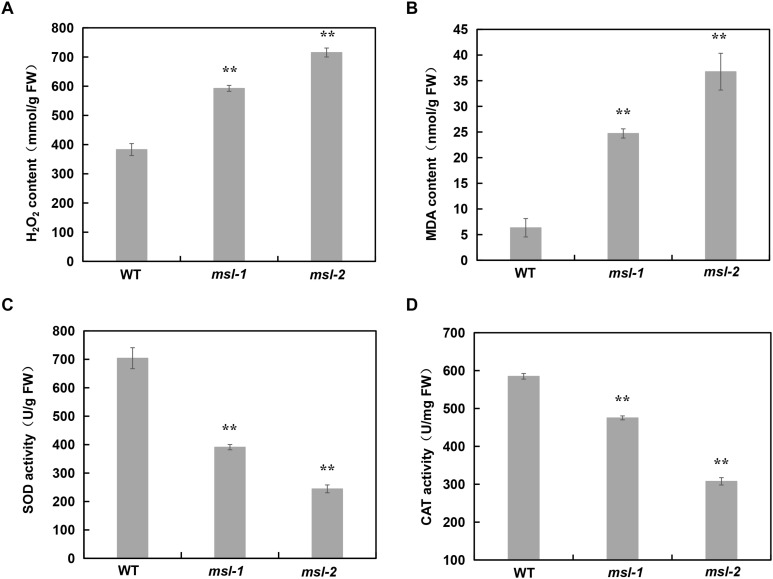
Analysis of ROS accumulation and relative expression of antioxidant enzymes in wild type and mutants. **(A)** H_2_O_2_ contents. **(B)** Malondialdehyde (MDA) contents. **(C)** SOD enzyme activity. **(D)** CAT enzyme activity. Error bars represent the standard deviations of three biological replicates. **, significant differences at *P* < 0.01 (Student’s *t*-test).

The metabolic disorder of reactive oxygen species (ROS), mainly including superoxide anion radical (O^2–^), hydrogen peroxide (H_2_O_2_), hydroxyl radical (OH), and nitric oxide (NO), can accelerate leaf senescence and lead to premature senescence of plants. Cell death is usually associated with intracellular accumulation of H_2_O_2_. Physiological indexes of the mutants *msl-1*, *msl-2* and wild type have been measured at tillering stage, and results showed that a large amount of H_2_O_2_ was accumulated in *msl-1* and *msl-2* (Figure 3A), which are consistent with the results of DAB staining (Figure 1G).

The content of malondialdehyde (MDA) usually reflects the degree of lipid peroxidation in plants, and indirectly reflects the degree of cell damage. Our data showed that MDA content in *msl-1* and *msl-2* was significantly higher than that in the wild type (Figure 3B). Plants produce a series of antioxidant enzymes to eliminate ROS damage to cells, such as catalase (CAT), superoxide dismutase (SOD), and peroxidase (POD) (Miller et al., 2010). SOD catalyzes superoxide anion radical (O^2–^) disproportionation to H_2_O_2_, and CAT catalyzes the decomposition of H_2_O_2_. Therefore, we also measured the activities of CAT and SOD in *msl-1*, *msl-2* and the wild type. Results showed that the activities of SOD and CAT in *msl-1* and *msl-2* significantly decreased compared with the wild type at tillering stage (Figures 3C,D), which caused the ROS accumulation in the mutant leaves.

### Fine Mapping of *msl-1* and *msl-2*

To identify the gene(s) underlying the mosaic spot lesion and leaf senescence phenotypes of the mutants, we generated F_2_ progenies from crosses between the japonica rice cultivar 02428 and the mutants *msl-1* and *msl-2*, respectively. All the F_1_ individuals did not show any mosaic spot lesions and had normal phenotypes as the wild type, indicating that the mutant phenotypes were genetically recessive. In the F_2_ progenies, the segregation ratios between normal plants and the plants with spot lesions statistically fitted to 3:1 (Table 2), following the canonical Mendelian segregation, indicating that a single recessive gene controls the mutant phenotypes of both the mutants.

**TABLE 2:** Genetic analysis of mutants *msl-1* and *msl-2.*

Combination	F_1_	F_2_			χ^2^ (3:1)	*P*-value
		Wide-type	Mutant-type	Total		
02428 × *msl-1*	Normal	1,012	316	1,328	1.028	0.311
02428 × *msl-2*	Normal	1,422	458	1,880	0.408	0.523

The mosaic spot lesion plants in the F_2_ progenies were used to map the target gene(s). A total of 256 pairs of InDel molecular markers were selected for polymorphism screening. Among them, 52 pairs showed polymorphism between mutants and 02428. The polymorphic markers differentiating *msl-1* from 02428 were identical with those between *msl-2* and 02428, implying that the mutant genes in *msl-1* and *msl-2* may be alleles of a single locus.

**FIGURE 4:**
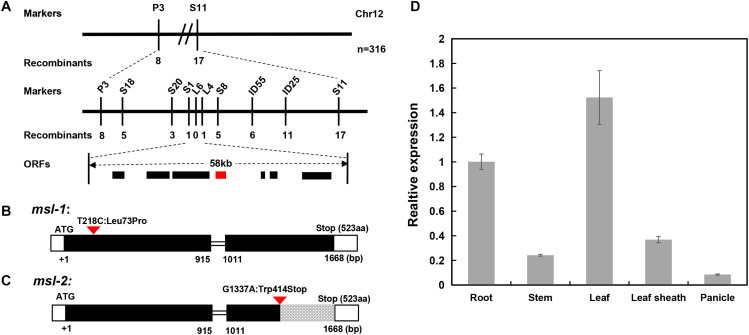
Map-based cloning of *msl-1* and *msl-2*. **(A)** The *msl-1* and *msl-2* loci were fine-mapped to 58kb region between Indel markers S1 and L4 on chromosome 12. **(B)** Gene structure of *LOC_Os12g16720* and the mutation site in *msl-1*. **(C)** Gene structure of *LOC_Os12g16720* and the mutation site in *msl-2*. The black rectangles represent exons and the red inverted triangle represents the mutation sites. **(D)** Analysis of *SL* gene expression in different tissues of WT. Error bars represent the standard deviations of three biological replicates.

We then conducted genetic linkage analysis on the mutant *msl-1*. After analyzing the polymorphic markers between the two parents, we found that most of the polymorphic markers distributed on chromosome 12, indicating that the target gene might locate on chromosome 12. Therefore, F_2_ individuals with obvious mosaic spot lesion phenotype were selected, and the polymorphism markers on chromosome 12 were used for the linkage analysis of *msl-1*. Results showed that there was a linkage phenomenon between the mutant phenotype and two markers designated as P3 and S11. We subsequently developed new molecular markers within the interval between P3 and S11, and the target gene was mapped between new markers S20 and S8, with a genetic distance of 1.41cM. In order to narrow down the target interval, another 3 pairs of polymorphic InDel markers between S20 and S8 were developed, and genotyping was performed on more F_2_ individuals. The target gene was finally located between InDel markers S1 and L4, with a physical distance of about 58 kb (Figure 4A).

### Both *msl-1* and *msl-2* Are Allelic Variants of the SL Gene

According to the RGAP website of the rice genome database, there are 7 annotated genes (Supplementary Table 3) in the 58 kb target region flanked by molecular markers S1 and L4. By sequencing all the candidate genes in the interval, we found that only *LOC_Os12g16720* has a SNP between sequences from *msl-1* and the wild-type JG30. Compared with JG30, there is a base substitution (T to C) at 218 nucleotide position (T218C) in the first exon of *LOC_Os12g16720* locus in *msl-1*, which causes an amino acid change from isoleucine to proline at 73rd amino acid position (Leu73Pro) (Figure 4B). To further verify the candidate gene, we amplified and sequenced the *LOC_Os12g16720* locus in F_2_ plants from the cross between 02428 and *msl-1*, and found that the T218C single-base alteration presented in all the F_2_ plants with mosaic spot lesion phenotype but not in the normal F_2_ plants. Based on nucleotide sequences and rice genome annotation information, *LOC_Os12g16720* is the previously reported *SL* (Sekiguchi lesion) gene, encoding a CYP71P1 protein of cytochrome P450 monooxygenase family, which has tryptamine 5-hydroxylase activity and catalyzes the conversion of tryptamine to serotonin (Fujiwara et al., 2010). Therefore, we concluded that *msl-1* is an allelic variant of the *SL* gene, with the T218C SNP conferring the mosaic spot lesion and leaf senescence phenotypes of the mutant.

Since the genetic mapping implied that the mutant genes in *msl-1* and *msl-2* may be alleles of a single locus, we then sequenced all the above-mentioned candidate genes in mutant *msl-2*, and found that there is only a single base mutation (G to A) at 1,337 nucleotide position (G1337A) in the second exon of the *LOC_Os12g16720* locus in *msl-2*, which caused a premature termination of protein translation (Figure 4C). Similarly, we sequenced the *LOC_Os12g16720* locus in F_2_ plants from the cross between 02428 and *msl-2*, and found that the G1337A single-base substitution presented in all the F_2_ plants with mosaic spot lesion phenotype but not in the normal F_2_ plants. Thus, the single base substitution in the *LOC_Os12g16720* locus confers the mosaic spot lesion phenotype of both mutants *msl-1* and *msl-2*. This conclusion has been supported by the recently published research on *ell1* (early lesion leaf 1) and *sl-MH-1*, which are LMM mutants from the japonica rice variety Wuyunjing7 and indica rice line Minghui 86 (MH86), respectively, at the *LOC_Os12g16720* locus (Tian et al., 2020; Cui et al., 2021).

To check the expression pattern of the *SL* gene, qRT-PCR analysis was performed using different tissues including roots, stems, leaves, leaf sheaths and spikelets of wild-type JG30 at booting stage. Results showed that the *SL* gene was constitutively expressed in all the tissues. However, the expression levels in root, leaf and leaf sheath were higher than in other tissues, which is consistent with the fact that the occurrence of mosaic spot lesion mainly concentrated on roots, leaves and leaf sheaths (Figure 4D).

### Bioinformatics Analysis of the Proteins Encoded by *msl-1* and *msl-2*

**FIGURE 5:**
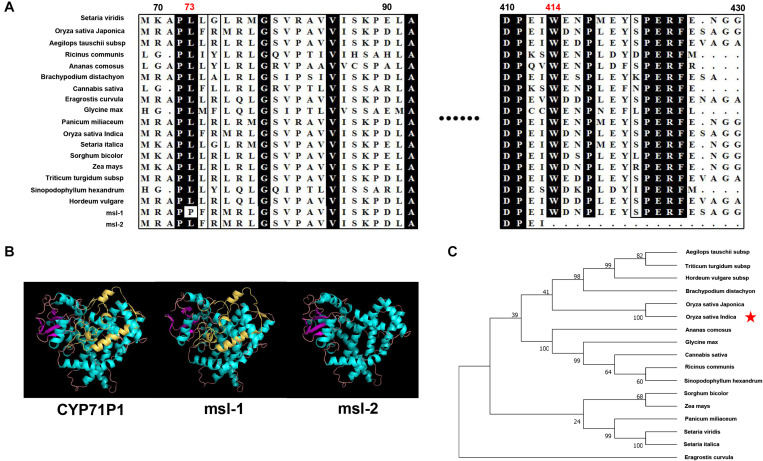
Bioinformatics analysis of target genes. **(A)** Protein sequence alignment of the CYP71P1 protein homologies among multiple species, both the mutation sites of msl-1 (at 73nd residue) and msl-2 (at 414th residue) are highly conserved. **(B)** Protein three-dimensional structure of CYP71P1, msl-1 and msl-2. **(C)** Phylogenetic tree analysis of the CYP71P1 homologies.

Since *msl-1* and *msl-2* are allelic variants of the *SL* gene that encodes the CYP71P1 protein, bioinformatics analysis on the CYP71P1-homologies was conducted. Multiple sequence alignment of target protein sequences from 17 species showed that the two amino acids (L73 and W414) are highly conserved among various species (Figure 5A). This should conform that the single nucleotide substitution (T218C) in *msl-1*, that causes the 73rd amino acid change (Leu73Pro), affects the biological function of the msl-1 protein, and resulted in the *msl-1* mutant phenotypes. Furthermore, since the G1337A SNP in *msl-2* causes premature termination of protein translation at the 414^*th*^ amino acid position (Figure 5A), the deduced msl-2 protein lacks the 109 aa-C-terminus compared with the wild type CYP71P1, this may make the *msl-2* show more serious mutant phenotypes than that of *msl-1*. The protein sequences of wild-type CYP71P1 and its mutants msl-1 and msl-2 were compared and analyzed by Swiss-model for predicting the three-dimensional structure of proteins. Deletion of the 109 aa-C-terminus in msl-2 led to the obvious conformational change of the protein (Figure 5B). However, the protein structures of msl-1 and WT CYP71P1 showed no significant difference (Figure 5B), which to some extent explained the significant difference in phenotypes of the two mutants. In addition, we used neighbor-joining method to build a phylogenetic tree, it indicated that the CYP71P1 and its homologous proteins in rice are closely related to monocots such as grasses (Figure 5C).

## Discussion

### Both *msl-1* and *msl-2* Belong to the Expanding Type of Lesion Mimic Mutants but Differ Largely in Severity

In this study, we identified two LMMs (*msl-1* and *msl-2*) with stable genetic traits which were obtained from indica rice cultivar JG30 treated by EMS. Although there are no obvious differences between the two mutants at seedling stage (Figures 1A,C), the phenotype differences appeared along with the development of the plants. At tillering stage, not only more mosaic spots appeared on leaves of *msl-2* but also the spot size was larger than that of *msl-1* (Figure 1D). At maximum tillering stage, the leaves of *msl-2* appeared chlorotic, the lower leaves gradually died, and the plants showed more obvious premature senescence than *msl-1* (Figure 1B). At mature stage, the leaves of *msl-2* were completely yellow and the whole plant dead but not for the *msl-1* mutant (Figure 1E). Thus, both *msl-1* and *msl-2* displayed mosaic spot lesion and premature senescence phenotypes but differed largely in severity.

Lesion mimic mutants have been divided into two types according to the pattern of spots formation: the initial type and the expanding type (Dangl et al., 1996). In this study, the location of mosaic spots of both *msl-1* and *msl-2* are uncertain, and along with the development of the leaves, the spots enlarged and even covered the whole leaf, which ultimately led to death of a whole leaf. Therefore, the *msl-1* and *msl-2* belong to the expanding mutants. It had been reported that lesion mimic spots could also be affected by a series of external environmental factors, such as temperature (Hoisington et al., 1982), humidity (Jambunathan et al., 2001), and light (Johal et al., 1995). The formation of mosaic spots on leaves of *msl-1* and *msl-2* was induced by light, which showed that shading could inhibit the occurrence of mosaic spots, and the spots reappeared after regaining normal light (Figure 1F).

### Different Modes of Mutation Lead to Different Mutant Phenotypes

**FIGURE 6:**
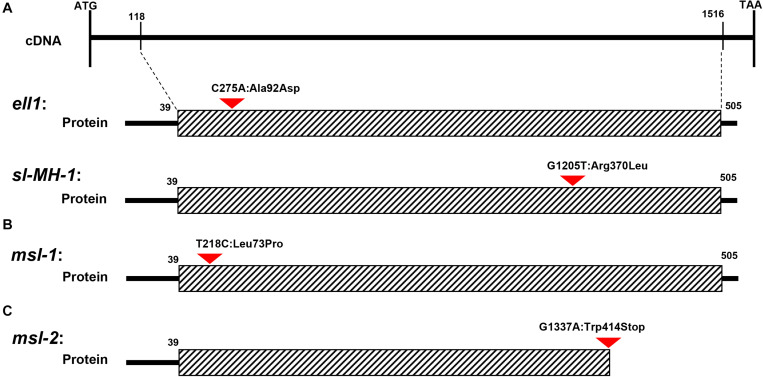
Schematic structure of CYP71P1 protein and the mutation sites in msl-1 and msl-2. The black dotted bordered rectangle indicates CYP71P1 the highly conserved P450 superfamily domain of wild type CYP71P1. **(A)** The mutation site of *ell1* and *sl-MH-1*, *ell1* had a single base substitution at 275th nucleotide position (C275A) and *sl-MH-1* also had a single base substitution at 1,205th nucleotide position (G1205T). **(B)** The mutation site of *msl-1*, with a base change from T to C at the 218th position. **(C)** The mutation site of *msl-2*, with the G-to-A single-base alteration, leading to premature termination of the protein translation.

The mutant phenotypes of *msl-1* and *msl-2* were controlled by a single recessive gene (Table 2). This study revealed that both the recessive genes underlying the mutant phenotypes of *msl-1* and *msl-2* are allelic variants of the *SL* gene, which encodes a CYP71P1 protein of cytochrome P450 monooxygenase family, with tryptamine 5-hydroxylase activity to catalyze the conversion of tryptamine to serotonin (Fujiwara et al., 2010). We showed that the differences of mutant phenotypes between *msl-1* and *msl-2* were due to different mutation sites and mutation types in the *SL* gene. This gene is composed of two exons and one intron, and coding a protein contained 523 amino acids. It was predicted that amino acids from positions 39–505 of this protein form a P450 domain (Figures 6A–C). Both mutants *msl-1* and *msl-2* had a single base mutation in this domain. The mutant *msl-2* has a G-to-A single-base alteration, leading to premature termination of the protein translation (Figure 6C); while *msl-1* was mutated from T to C at 218 nucleotide position (T218C) in the first exon of *SL*, which cause an amino acid change from isoleucine to proline (Leu73Pro) (Figure 6B). Recently, Cui et al. (2021) have cloned another allelic mutant (*ell1*) of *SL*, which has a single base mutation at 275 nucleotide position (C275A) in the first exon and changed an amino acid from alanine to aspartic acid at the 92nd position of protein sequence (Figure 6A). Interestingly, additional mutants at the *SL* locus have been generated from the indica rice line MH86 by tissue culture (*sl-MH-1*) and by ^60^Co ∼γ-ray radiation (*sl-MH-2* and *sl-MH-3*), respectively (Tian et al., 2020). These three mutants also spontaneously exhibit orange-colored lesions on leaves. A G to T mutation was found at 1,205 nucleotide of *SL* ORF, which leads to the 370 Arg mutated to Leu in *sl-MH-1*, while *sl-MH-2* and *sl-MH-3* carry C85 and A1420 deletion in the *SL* coding region, respectively, whose phenotypes have not been systematically studied (Tian et al., 2020). After comparison, we found that *ell1*, *sl-MH-1*, and *msl-1* have similar phenotypes. Based on the differences in three-dimensional structure of the proteins, we speculated that the mutation of *msl-2* which was in the key structural domain and causing pre-termination of the protein translation, resulted in the complete destruction of the protein function, while a single amino acid mutation occurred in *msl-1* or *ell1*, the protein may still retain some function. Therefore, the difference of mutation sites and mutation types (SNP-caused single amino acid change and SNP-caused early termination of translation) led to the different phenotypes in severity between *msl-1* and *msl-2*.

### The Mutation of CYP71P1 Leads to Premature Senescence

Plant cytochrome P450 (CYP450) is a class of enzymes that have been classified as superfamily (Nebert et al., 1989). CYP450 is named because its reduced state can combine with CO and has the strongest absorption spectrum at 450 nm (Denisov et al., 2005). CYP450 is involved in the biosynthesis and catabolism of many substances in organisms, including various fatty acid conjugates, plant hormones, secondary metabolites and defense compounds. Because of its involvement in various physiological and biochemical reactions, CYP450 is called universal biocatalyst. It also plays an important role in plant growth and development (Bak and Feyereisen, 2001), as well as in the response to biotic and abiotic stresses (Elzaki et al., 2019).

CYP71P1, a member of the cytochrome P450 monooxygenase family, has tryptamine 5-hydroxylase activity, and catalyzes the conversion of tryptamine to serotonin (Fujiwara et al., 2010). Serotonin is known as neurotransmitter which widely distributed in mammalian tissues, especially in cerebral cortex and synapses. In plants, serotonin is found in a range of species and plays an important role in various physiological functions (Roshchina, 2001). Serotonin synthesis involves two steps: tryptophan decarboxylase (TDC) catalyzes the conversion of tryptophan (Trp) into tryptamine, then tryptamine is further catalyzed to serotonin by tryptamine 5-hydroxylase (T5H) (Kang et al., 2007). It has been shown that serotonin is involved in slowing down leaf senescence (Kang et al., 2009), this might explain why *msl-1* and *msl-2* showed premature senescence phenotype. The mutant *msl-2* leaves aged rapidly after flowering and faded to yellow, and the leaves died at the mature stage (Figure 1E), indicating that the loss-of-function of CYP71P1 led to the occurrence of early senescence. Moreover, the different mutant types resulted the different expression levels of senescence-related genes, and eventually lead to different degrees of premature phenotype.

**FIGURE 7:**
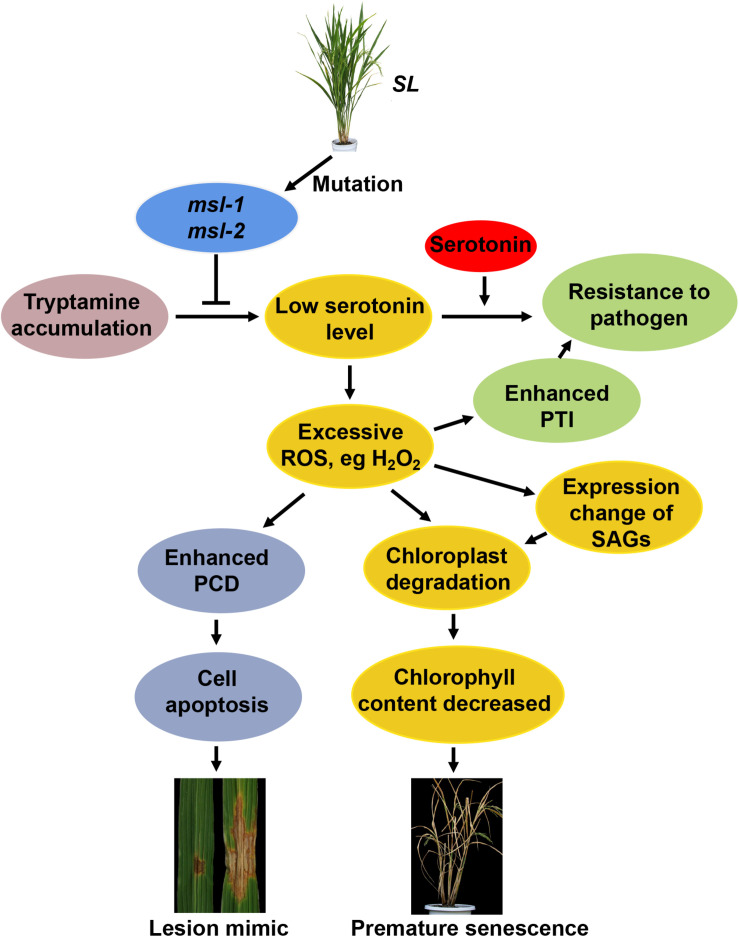
Proposed working model for *SL*-involved rice leaf senescence and cell death. The conversion of tryptamine to serotonin is catalyzed by CYP71P1 encoded by *SL* (Sekiguchi lesion) gene. Mutations of *SL* affect or block the catalytic process, accompanied with accumulated tryptamine and the low level of serotonin. Exogenous applied serotonin enhances resistance to *Magnaporthe grisea* (*M. grisea*) in the *sl* mutant. Low level of serotonin causes excessive reactive oxygen species (ROS) accumulation in the *sl* mutants, triggering programmed cell death (PCD)-mediated cell apoptosis, resulting in lesion formation. Excessive ROS also contribute to activation of pathogen-associated molecular patterns (PAMPs)-triggered immunity (PTI) to plant pathogens like *M. oryzae* and *Xanthomonas oryzae* pv. *oryzae*. ROS can function as signaling messengers to induce chloroplast degradation directly or by regulating the changes of senescence-associated genes (SAGs), resulting in the premature leaf senescence phenotype.

Based on our results and those findings reported previously, we proposed a working model for the role of the *SL* gene in rice leaf senescence and cell death (Figure 7). In rice, the conversion of tryptamine to serotonin is catalyzed by CYP71P1 encoded by *SL*. Once *SL* mutates, the catalytic process will be affected or blocked, accompanied with the accumulation of tryptamine and the low level of serotonin. It has been shown that serotonin is involved in the immune response of plants, and exogenous applied serotonin enhances resistance to *Magnaporthe grisea* (*M. grisea*) in the *sl* mutant (Fujiwara et al., 2010). In addition, the serotonin is acknowledged to be a kind of strong antioxidant compounds by scavenging ROS (Huether et al., 1997), which is in agreement with excessive ROS accumulation in the *sl* mutants (Cui et al., 2021; Figure 3). Excessive ROS accumulation triggers off programmed cell death (PCD)-mediated cell apoptosis, causing the lesion formation in rice (Cui et al., 2021). Moreover, ROS could also contribute to activate the pathogen-associated molecular patterns (PAMPs)-triggered immunity (PTI) responses, and thus the increased resistance to *M. oryzae* and *Xanthomonas oryzae* pv. *oryzae* (Arase et al., 2001; Tian et al., 2020). Additionally, ROS can function as signaling messengers to induce chloroplast degradation directly or by regulating the changes of senescence-associated genes (SAGs) (Gechev et al., 2006; Mao et al., 2017), and the degradation of chlorophyll further leads to decrease of chlorophyll content in the leaves of *sl* mutants, ultimately resulting in the premature leaf senescence phenotype (Figure 7).

In short, the novel mutants *msl-1* and *msl-2* identified in this study represent two new alleles of the *SL* gene which encodes a cytochrome P450 monooxygenase (CYP71P1), distinct from the previously reported *sl*, *sl-MH-1*, and *ell* alleles. Our results indicate that the different mutations in CYP71P1 could lead to different phenotypes in severity of both mosaic spot lesion and premature senescence. The findings might provide new insights into the regulation of chloroplast development and programmed cell death pathways during rice leaf senescence.

## Data Availability Statement

The original contributions presented in the study are included in the article/Supplementary Material, further inquiries can be directed to the corresponding author/s.

## Author Contributions

KZ and CW conceived and designed the research. YZ, JX, FW, YT, and ZW performed the experiments. YZ, JX, and ZJ participated in data analysis. YZ and KZ wrote the manuscript. All authors contributed to the article and approved the submitted version.

## Conflict of Interest

The authors declare that the research was conducted in the absence of any commercial or financial relationships that could be construed as a potential conflict of interest.

## Footnotes

1 http://rice.plantbiology.msu.edu

2 http://primer3.ut.ee

3 https://swissmodel.expasy.org/

## Supplementary Material

Supplementary Table 1 Primers used for real-time quantitative PCR analysis.

Supplementary Table 2 Molecular markers for fine mapping of *msl-1* and *msl-2.*

Supplementary Table 3 The candidate genes in the mapped 58 kb region.
