# Supplementary material for: Mutation Types of CYP71P1 Cause Different Phenotypes of Mosaic Spot Lesion and Premature Leaf Senescence in Rice
Source: Front Plant Sci. 2021 Mar 23;12:641300. doi: 10.3389/fpls.2021.641300 (PMC8021961; doi:10.3389/fpls.2021.641300)
Supplement: Supplementary Table 1 — Primers used for real-time quantitative PCR analysis. [file Table_1.DOCX]

Supplementary Material

# Supplementary Tables

**Supplementary Table 1.** Primers used for real-time quantitative PCR analysis

| Primer | Forward primer (5’-3’) | Reverse primer (5’-3’) |
| --- | --- | --- |
| *OsActin* | TGGCATCTCTCAGCACATTCC | TGCACAATGGATGGGTCAGA |
| *SGR* | AGGGGTGGTACAACAAGCTG | GCTCCTTGCGGAAGATGTAG |
| *Osh36* | GCACGGAGGCGAACGA | TTGAGCGGTAGCACCCATT |
| *Osl85* | GAGCAACGGCGTGGAGA | GCGGCGGTAGAGGAGATG |
| *V1* | TGGAGGTCGGGACAGAGGA | CGAGGAGCACCACCATCAC |
| *V2* | GCAGCAGATCCGTGATTACA | GCTGCTCCTTGAATGTCCAC |
| *OsDVR* | CAGGTCGAGACCGTCAAGAAC | ATGACCTGGATCGGCACCTTG |
| *OsCHLH* | AACTGGATGAGCCAGAAGAGA | AAATGCAAAAGACTTGCGACT |
| *OsPORA* | ATGGCTCTCCAAGTTCAG | TGGCTCACGCTAAGGAAC |
| *OsPORB* | CCGCAAGGAGGGAGCGGTG | CCCTCTTGGTGCTAAGGCCG |
| *psaA* | GGAGGTGGCGAGTTAGTA | GATTTGCTTTATCGGGTAT |
| *psbA* | TATGGGTCGTGAGTGGGA | TTATGCTCTGCCTGGAAT |
| *rbcL* | CTTGGCAGCATTCCGAGTAA | ACAACGGGCTCGATGTGATA |
| *rbcS* | CAGCAATGGCGGCAGGAT | AGGGCACCCACTTGGAACG |
| *cab1R* | AGATGGGTTTAGTGCGACGAG | TTTGGGATCGAGGGAGTATTT |
| *cab2R* | TGTTCTCCATGTTCGGCTTCT | GCTACGGTCCCCACTTCACT |
| *rpoA* | CGCATCAATTTGCGTCAAAG | GTTAGCTATAGGTTGTGCCGTATCA |
| *rpoB* | CAAGTTTTCGGAGCCGAGAT | GCTAAAGATCCAGTAAGTCCAACG |
| *OsSL* | AAGGGAGAGGAGCTTAGTGAGG | AGCTCCAGCCTCCAGGTTACTTAG |

**Supplementary Table 2.** Molecular markers for fine mapping of *msl-1* and *msl-2*

| Primer | Forward primer (5’-3’) | Reverse primer (5’-3’) |
| --- | --- | --- |
| P3 | TGTATGCCTTTGATTCGTAGGA | CCAAACACATGAAAACGGCG |
| S18 | AAGCTACTCACTGGATCCGC | CGATCTGGACTGTTAATCTGCA |
| S20 | GGTCGCGAGGTTTCTTGATG | GAGGGGAAAACACGTTGACC |
| S1 | TGGAGAGAGAGTAGGGGAGG | CGCCGTAATCATTTCCATTTCC |
| L6 | TGCCCACTTTACAGTCACAGA | CCTCATGCATGTGTCCAAACA |
| L4 | CTTTGAACCTCACCCCGTTG | GCGGTAGGGTTTCAGAGGTA |
| S8 | TTGCCCCATCTCTACCGTTT | TGGCGCCCTATAAATCTGGT |
| ID55 | TGGCGCCCTATAAATCTGGT | AGGTCTGATCACTCCCTCCA |
| ID25 | GCAGCAAGGAATCCAAGGAG | AGACAGAGAGTATGCGTGCA |
| S11 | CGCATGAGGAAAAGAGACAGG | AGTGACACTGTTTGAGCATGT |

**Supplementary Table 3.** The candidate genes in the mapped 58 kb region

| Gene | Gene ID | Annotation |
| --- | --- | --- |
| ORF1 | LOC_Os12g16680 | Expressed protein |
| ORF2 | LOC_Os12g16690 | Zinc finger, C3HC4 type domain containing protein, expressed |
| ORF3 | LOC_Os12g16710 | Retrotransposon protein, putative, Ty1-copia subclass, expressed |
| ORF4 | LOC_Os12g16720 | Cytochrome P450 71A1, putative, expressed |
| ORF5 | LOC_Os12g16730 | Retrotransposon protein, putative, Ty1-copia subclass, expressed |
| ORF6 | LOC_Os12g16740 | Retrotransposon protein, putative, Ty1-copia subclass, expressed |
| ORF7 | LOC_Os12g16750 | Retrotransposon protein, putative, unclassified, expressed |
